# Annotations

**Published:** 1890-09-13

**Authors:** 


### Annotations.

Miss Layard and the Honourable Lady Welby are evidently of opinion that "the proper study of mankind is man"; and with the view of getting man studied with due impartiality they attacked the anthropologists last week at the British Association in their own stronghold. It is well known that Darwin, Professor Laycock, Pinel, Dr. Maudsley, and many other persons have maintained the doctrine of "reversion" in man and animals. Darwin says, "When a structure is arrested in its development, but still continues growing until it closely resembles a corresponding structure in some lower and adult member of the same group, it may, in one sense, be considered as a case of reversion. . . The simple brain of a microcephalous idiot, in as far as it resembles that of an ape, may in this sense be said to offer a case of reversion." Nothing of the kind, argues Miss Layard; and she proceeds to show that on this point Darwin may be confuted out of his own mouth. For the very first words which he employs in introducing the subject of "Reversion" in the *Descent of Man*, p. 36, are these: "Many of the cases to be here given might have been introduced under the last heading." Now the last heading is "Arrests of Development." Miss Layard considers it far more likely that the true explanation of all cases of the kind under discussion is arrest of development and not reversion. She quoted the statement that "the proportion of blood of any one ancestor after twelve generations is only one in 2,048." If that be indeed so, what must be the proportion of ancestral ape's blood in any living man at the present time to that of the original Simian from which he took origin three or four millions of years ago? Dr. Garson spoke at some length in reply, and remarked with very obvious truth that "the question raised involved a great many complicated problems." Several members of the audience thereupon were inclined to believe that the orthodox anthropologists were routed, and asked if it were to be taken for granted that there was really no proper evidence in favour of reversion to former types. Dr. Garson said there were decided instances of reversion to a more primitive type of man. He said it, but he did not prove it; and Miss Layard most decidedly turned the tables on the orthodox "Reversionists" in her reply. She said "that the down on the human body and the long hairs on a delicate child were cases not of reversion but of arrested development. These hairs were not at all similar to the fur of the ape. If the fur on the human body was a vestige of the more perfect fur of the ape, on what animal originally did the down appear which had developed into the fur of the ape? One might suppose that the man with his down was the original progenitor of the ape." Nobody can deny that a new light is thrown upon the subject by Miss Layard's intellectual acuteness. If she succeeds in seriously modifying the general tendency of Darwinism, she will have to be placed in an intellectual rank above that even of lady Senior Wranglers.

**Miss Layard Turns the Tables.**

Last week died Dr. Mathews Duncan, one of the physicians to St. Bartholomew's Hospital, and to certain members of the Royal Family. Her Majesty the Queen sent a telegram of condolence to Mrs. Duncan and her family when she heard the intelligence of Dr. Duncan's death. Dr. Duncan was not old —only 64 years of age—at the time of his death. He died suddenly, of syncope, due to heart disease of some standing. All those who had known Dr. Duncan long will feel the grief which springs from a sense of personal loss. He was not every man's friend; but he was the friend of all who knew him well enough to be able to appreciate his character. He was, before all things, "a plain, blunt man," who loved the truth. He had not "the manners of the Court," if by that phrase is meant a supple obsequiousness which is always proclaiming its willingness to be annihilated a hundred times an hour for the Court's pleasure. But such are not the manners of the Court in these times, thanks to the truly royal simplicity of Her Majesty the Queen, and to the equally simple and manly frankness of the Prince of Wales; thanks also, and quite as much, to the still bright and youthful lady, the Princess of Wales, whom all men love and of whom no woman is jealous. Dr. Duncan was a Court physician who never ceased to be the same straightforward and honest man that won the hearts of Edinburgh students nearly a quarter of a century ago by his outspoken contempt for baseless theories and his uncompromising adherence to plain, demonstrable, and intelligible facts. The line of practice which he chose for himself is one which lends itself with peculiar facility to the arts of the professional pretender, and the legally qualified knave. Nobody knew this better than Dr. Duncan, and it is not improbable that his sometimes almost offensive directness of manner may have been due to his honest dread of even appearing to give countenance to views and opinions which had no foundation in fact. The present writer has more than once known him give mortal offence and lose valuable patients because he has steadfastly denied the existence of disease which had for years been affirmed by other medical men, to their very great profit. Dr. Duncan might have his peculiarities, and, being mortal, even his faults. But he was a man who, taken all round, had few equals in the medical profession, and perhaps no superior. The salt of his honest simplicity was much needed among specialists of his own kind; and it will be greatly missed. Of him, it may be truly said, as of a still greater man, that "he has fought a good fight," and preserved unimpaired an honest conscience, and a truth-loving mind. Would that there were more such doctors and such men!

**The Late Dr. Mathews Duncan.**

"The German women," says a writer in the *Daily Graphic*, "are not beautiful, but they are loveable and sweet. They do not seem to trouble themselves about their 'rights,' but appear to be very contented and happy, even without votes. The men treat them with courtesy and tenderness, but with none of that exaggerated deference that one sees among the more petticoat-ridden nations nearer home. The Germans are women lovers, not women worshippers; and they are not worried by any doubts as to which sex shall rule the state, and which stop at home and mind the children. The German women are not politicians, and mayors, and county councillors: they are housewives." The writer is, of course, a man, and expresses sentiments which commend themselves to probably a large number of his fellow-countrymen. He is, no doubt, very well pleased with them himself. But after all, what do his statements amount to? Let it be granted that all he says is true—how does it affect the question of what may be called the "woman's" movement in this country? It does not affect it at all, any more than a description of the German men would affect the question of the rights and character of the men of this country. The German women have their own peculiar characteristics; and they must play their part in the world with such instruments as nature has furnished them with. English women have their characteristics too, and if they are different from those of the German women, who is responsible; and why is the fact to be regretted, if it is to be regretted? Is not this the real state of the case with us in this country: that we need more brain capacity, more kindly feeling and more sympathy in every department of public business which we have to undertake? If here and there we find a woman who has brain capacity for such business, and if that capacity be associated with kindly feeling and intelligent sympathy, why, in the name of common or uncommon sense, should not such a woman be asked to take a share in the public business? Nobody surely is so blind as not to see that men by themselves grievously bungle all sorts of public affairs, and never by any chance carry them out more than approximately well. The really sensible man, instead of warning off critical and capable intellects, is always on the look-out for their assistance. What, after all, is wanted is that the world's business shall be done in the most efficient way. If an ass, or an elephant, or a chimpanzee had better brains than man for certain parts of life's work, what a fool man would be not to secure the help of such brains! Is there a son, or a brother, or a husband anywhere that does not know by experience that in some of the departments of domestic life woman is superior to man? What is municipal or national life but domestic life in the large? Is it not reasonable, therefore, to suppose that in the largest as well as in the smallest affairs of life there may be departments and occasions where woman is the fittest person to possess authority or to give advice? The average Englishwoman is second to none as a mother of children and as a housewife. If, in addition to these excellencies, she has a capacity for dealing with the poor, or with school children, or even with the larger political affairs of the nation, is she not all the more to be honoured, and is not her help all the more to be sought?

